# Exploring the Influence of the Edmonton Symptom Assessment System Implementation in Palliative Care Patients: A Systematic Review

**DOI:** 10.7759/cureus.70914

**Published:** 2024-10-06

**Authors:** Hassan R Alsuliman, Sukaynah A Alsaigh, Faisal A Habib, Maied Z Alshehery

**Affiliations:** 1 Department of Palliative Medicine, King Fahad Medical City, Riyadh, SAU

**Keywords:** assess, care, esas, palliative, symptom

## Abstract

One of the most essential elements of providing high-quality palliative care is good symptom control, which is guided through an assessment of the evolution of the patient's level of distress over time. The Edmonton Symptom Assessment System (ESAS) was created to measure diverse symptoms in palliative care patients. It is a user-friendly, validated, and reliable multi-item instrument. Globally, ESAS is extensively used to direct daily clinical care and foster communication across multidisciplinary teams, ensuring coordinated patient management, facilitating referrals to specialized programs, and evaluating the quality of care. In this systematic review, we aim to evaluate the influence of using ESAS on symptom assessment and control among palliative care patients and settings.

We have employed both manual and electronic search strategies among databases to determine relevant studies. This systematic review included original studies published between 2013 and 2023 that implemented the ESAS as part of palliative care for adult patients with terminal illness or advanced disease. Studies recruiting pediatric patients, case reports with limited sample sizes and no descriptive statistics, and nonhuman or laboratory studies were excluded. The ROBINS-I (Risk Of Bias In Non-randomized Studies-of Interventions) tool was used to assess the quality of the included studies, which assessed the methodological quality and potential risk of bias in non-randomized clinical studies.

We included eight studies that recruited a total of 3184 patients. Fatigue followed by pain were the symptoms with the highest score on ESAS among all the studies, while the lowest score was recorded for nausea and dyspnea in most of the studies. ESAS scores showed improvement in the follow-up visits. Two of the included studies reported satisfaction with the utilization of the ESAS tool. As stated in our results, it can improve the overall quality of life for patients receiving palliative care by assisting healthcare professionals, family members, and caregivers in methodically assessing and treating symptoms over time. By fostering better communication among care teams and involving caregivers in the process, the use of ESAS promotes a more patient-centered approach to care, ensuring that both the patient's needs and the perspectives of their loved ones are considered.

## Introduction and background

Across the globe, the population is aging and suffering from chronic diseases. It is critically important for the provision of patient-centered end-of-life care as the prevalence of chronic diseases has significantly increased due to longer life expectancy. Although they do not jeopardize life expectancy, chronic diseases have a substantial impact on a person's health and medical demands. Dementia, cardiovascular and respiratory diseases, cancer, and other more severe life-limiting chronic disorders are on the rise and have altered the pattern of death. Presently, chronic diseases are the main cause of death in older people after a long period of disability and decline [1]. The need to reevaluate the objectives of medicine is being driven by the rising understanding of the complex care requirements of patients with life-limiting diseases and their families. Due to changes in demographics, medical technology, and diseases over the past 10 years, the landscape of palliative and end-of-life care has undergone significant change. As a result, an increasing number of patients who suffer from chronic, progressive disorders require palliative care [2].

Palliative medicine is a type of specialist healthcare for those with life-threatening diseases. A serious illness involves a significant mortality risk, impairs function or quality of life, or is burdensome in terms of symptoms, treatments, or caregiver stress [3]. The purpose of palliative care is to enhance the quality of life for patients through expert symptom management, psychosocial support, patient-physician communication, facilitating treatment planning, and extensive family consultations regarding end-of-life care. Incorporating a multidisciplinary team and a complete strategy that tackles the physical element as well as the emotional, spiritual, and practical realms is essential to providing palliative care. During the past few decades, there has been a growing understanding of the advantages of combining palliative care with curative therapies [4]. The number of people who need palliative care at the end of their lives is predicted to be 20 million globally each year, with adults above 60 years comprising 69% of this group and children accounting for 6% [1].

The benefits of palliative care in providing patients with complete care through skilled symptom management are becoming clearer. Palliative care has been found to enhance the quality of life for cancer patients by treating a variety of symptoms, such as pain, breathlessness, nausea, and others [5]. One of the most essential elements of providing high-quality palliative care is good symptom control. The effectiveness of palliative care programs' activities targeted at lowering physical and psychological discomfort must be documented to guarantee high-quality care. This is possible by tracking the evolution of the patient's level of distress over time. Several evaluation tools are available; however, some multidimensional surveys are complicated and demand too much of seriously ill patients concerning time and concentration. The Edmonton Symptom Assessment System (ESAS) was created to measure diverse symptoms in palliative care patients. It is a user-friendly, validated, and reliable multi-item instrument [6]. Patients can self-report their symptom severity using the ESAS. Bruera and colleagues created it initially in 1991 to measure the symptom burden of patients with advanced cancer in an inpatient palliative care unit [5].

The ESAS is a 10-item, validated symptom assessment tool that evaluates general well-being, seven physical symptoms (pain, fatigue, nausea, drowsiness, shortness of breath, appetite, and insomnia), and two emotional symptoms (depression and anxiety). It has been translated and utilized for symptom evaluation in numerous countries throughout the world since its initial publication. In both clinical and research contexts, the ESAS is frequently used for symptom screening and longitudinal monitoring [7]. Globally, ESAS is extensively used to direct daily clinical care and foster communication across multidisciplinary teams, ensuring coordinated patient management, facilitating referrals to specialized programs, and evaluating the quality of care. During this busy time in the medical field, when efficiency and time savings are highly valued, a basic 10- to 12-item symptom assessment tool, like the ESAS, can assist clinicians in identifying distressing symptoms that are affecting patients' quality of life and potentially their ability to continue cancer treatments [8].

Research studies in the literature demonstrate that ESAS is a widely used tool in palliative care to assess and monitor patients' symptoms and general health. ESAS incorporates the patient's perspective by directly asking/involving them to assess their symptoms and health status while providing a standardized way to measure and quantify symptoms. Using ESAS in routine clinical practice, healthcare providers can monitor and note changes in patient's symptoms and health over time, which can help tailor treatment plans and interventions when needed. Moreover, the ESAS tool's validity and reliability are optimal, as reported in the literature. However, in this systematic review, we aimed to evaluate the influence of using ESAS on symptom assessment and control among palliative care patients and settings.

## Review

Methods

Inclusion and Exclusion Criteria

Our objective was to assess the influence of the utilization of ESAS on palliative care patients. Consequently, we included original studies that implemented the ESAS as part of palliative care for terminally ill or advanced disease patients. Studies recruiting pediatric patients were excluded. Moreover, case reports with limited sample sizes and no descriptive statistics were also excluded from this review. Other exclusion criteria were nonhuman or laboratory studies, non-original investigations or incomplete studies, abstract-only articles, protocols, theses, and articles that were not published in English or had no available English information.

Search Strategy

The following search terms were used: (ESAS OR Edmonton Symptom Assessment Scale OR Instrument Development OR Edmonton Symptom Assessment System OR Symptom Assessment) AND (Palliative Care Patients OR Palliative Medicine OR Palliative Treatment OR Palliative Therapy OR Palliative Care Unit OR Hospice and Palliative Care Nursing) AND (Symptom Management OR Patient Comfort OR Quality of Life).

**Figure 1:**
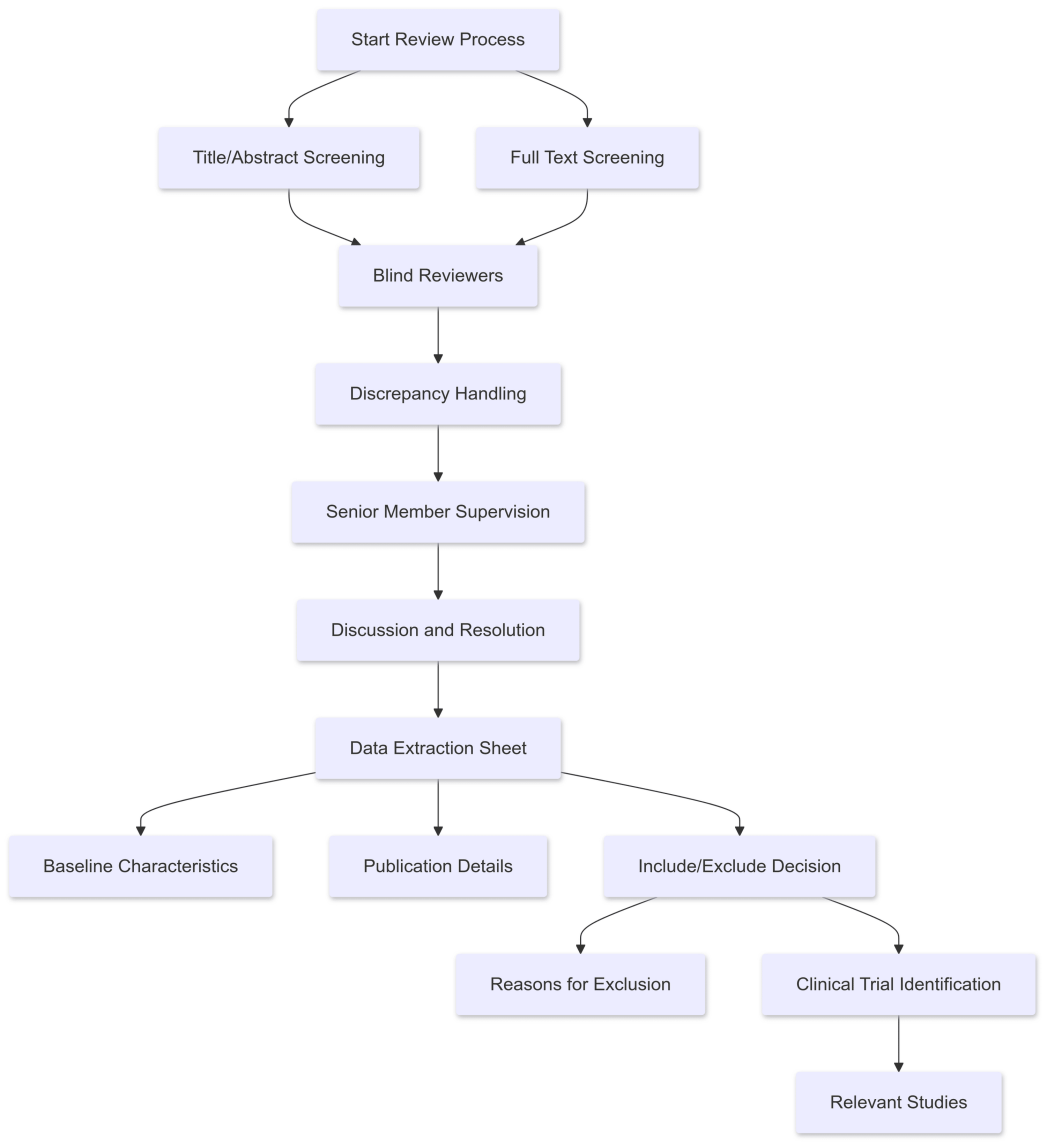
Flowchart illustrating the steps of screening and extraction of relevant studies

The databases searched included PubMed, Google Scholar, Web of Science, and ScienceDirect. Our search was restricted to the titles and abstracts of the search results to ensure that all relevant studies were included. All results were then saved to an EndNote library, where we identified and removed duplicates from the different databases. Additional manual searches were conducted on the reference lists of the included studies, relevant reviews, and the "similar articles" section in PubMed to identify any missed studies. Throughout all stages, we adhered to the Preferred Reporting Items for Systematic Reviews and Meta-Analyses (PRISMA) guidelines. Screening and extraction are illustrated in Figure 1.

Quality Assessment

We extracted information from the included studies regarding the potential risk of bias in these studies. To assess the quality of observational studies, we used the ROBINS-I (Risk Of Bias In Non-randomized Studies-of Interventions) tool, which assessed the methodological quality and potential risk of bias in non-randomized clinical studies [9]. This tool assesses the risk of bias in the following domains: confounding, selection of participants, classification of interventions, deviation from interventions, missing data, measurement of outcomes, and selection of reported results.

Results

Search Results

**Figure 2:**
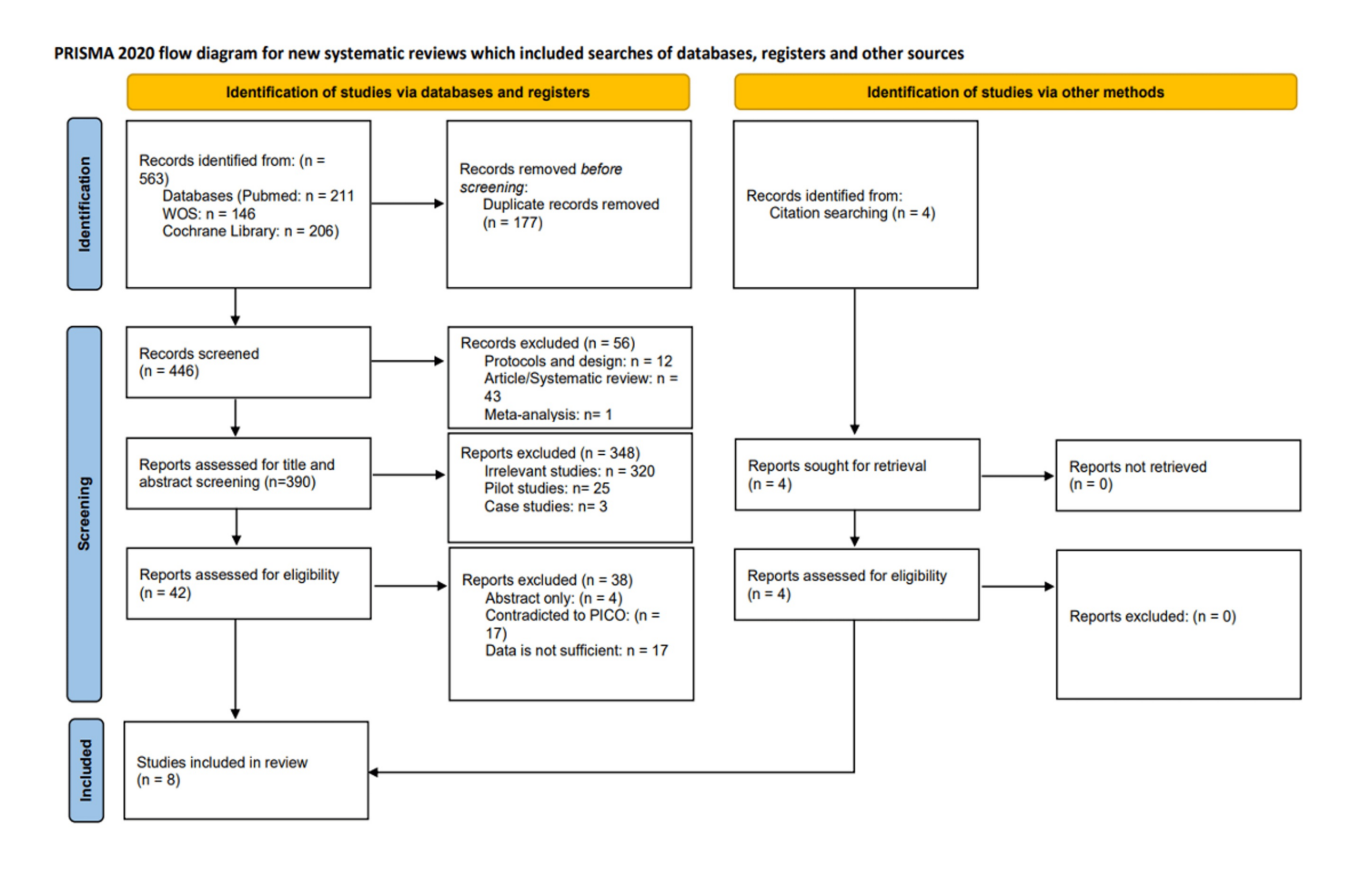
PRISMA flow diagram of study selection PRISMA: Preferred Reporting Items for Systematic Reviews and Meta-Analyses; WOS: Web of Science; PICO: Population, Intervention, Comparison, Outcome.

We conducted the search strategies as described above and identified a total of 446 citations, which were then reduced to 390 after removing duplicates. After screening titles and abstracts, only 42 citations were considered eligible for the next steps. Full-text screening narrowed down the number to eight articles that matched our inclusion and exclusion criteria. Figure 2 shows the detailed search strategy and screening process [10].

Results of the Quality Assessment

The quality assessment of the included studies revealed that four studies had a low to moderate risk of bias, three studies had a risk of bias, and one study had a critical risk of bias. None of the studies were found to have excellent or unsatisfactory results. The detailed results of the quality assessment according to the ROBINS scale are illustrated in Table 1.

**Table 1:** Quality assessment of the included studies using ROBINS-I tool ROBINS-I: Risk Of Bias In Non-randomized Studies-of Interventions.

Author	Confounding bias	Selection bias	Classification bias	Interventions bias	Missing data bias	Measurement bias	Reporting bias	Bias
Shin et al. [11]	Serious	Moderate	Moderate	Moderate	Moderate	Moderate	Low	Serious
Yokomichi et al. [12]	Moderate	Critical	Low	Low	Moderate	Moderate	Low	Critical
Kim et al. [13]	Low	Moderate	Low	Moderate	Low	Low	Moderate	Low
Hui D et al. [7]	Low	Moderate	Low	Low	Low	Low	Low	Low
Azhar et al. [14]	Serious	Serious	Low	Low	Low	Low	Low	Moderate
Brooks et al. [5]	Moderate	Serious	Moderate	Low	Low	Moderate	Low	Moderate
Delgado-Guay et al. [15]	Serious	Moderate	Low	Low	Low	Serious	Low	Serious
Wangnamthip et al. [16]	Serious	Serious	Low	Low	Serious	Low	Low	Serious

Characteristics of the Included Studies

We included eight studies [11-16] that recruited 3184 patients and were published between 2014 and 2021. The total population comprised 51.54% males and 48.45% females. The included studies were observational. Regarding the geographical distribution of the included studies, the majority were from the United States; three were multicenter studies, and one study was conducted in Thailand. All the baseline characteristics of these studies are shown in Table 2. The sample sizes of the included papers vary, likely due to the specific objectives and design of the study.

**Table 2:** Baseline characteristics of the included studies NR: Not reported.

Study	Country	Year	Study design	Study period	Total participants	Mean age	Gender (M/F)	Patient condition
Shin et al. [11]	USA	2014	Retrospective observational study	1-9-2003 to 31-8-2008	610	58.9 years	53%/47%	Cancer or delirium
Yokomichi et al. [12]	Multicenter	2015	Observational study	2-2013 to 6-2014	292	65 ± 12 years	64%/36%	Cancer
Kim et al. [13]	USA	2015	Retrospective observational study	2013	278	60 ± 13 years	50%/50%	Advanced cancer
Hui et al. [7]	Multicenter	2017	Prospective observational study	8-12-2011 to 30-4-2014	796	57 years	52%/48%	Cancer
Azhar et al. [14]	USA	2018	Retrospective observational study	1-9-2013 to 31-8-2014	544	60 years	51.7%/48.3%	Cancer
Brooks et al. [5]	USA	2019	Mixed methods	6-2018 to 12-2018	189	57.8 ± 14.7 years	44.97%/55.03%	Cancer
Delgado-Guay et al. [15]	Multicenter	2021	Observational study	13-3-2013 to 16-3-2016	325	Median age: 58 years	40%/60%	Cancer or chronic illness
Wangnamthip et al. [16]	Thailand	2021	Prospective observational study	1-2018 to 12-2018	150	58 years	56.7%/43.3%	Cancer

Study Outcome Measures

The symptom burden among patients was assessed through ESAS. Shin et al. reported the highest mean score of 6.5 for fatigue, while the lowest was reported for nausea at 2.2 [11]. Similarly, Yokomichi et al. reported the highest mean for fatigue 2.0 ± 2.6 and the lowest 0.5 ± 1.4 for nausea, while the total score in their study was 14.5 ± 21.3 [12]. Results of a study by Kim et al. also demonstrated an increased median score of six for fatigue and the lowest score of one for nausea, with a total median score of 39 [13]. In a study by Brooks et al., ESAS scores were assessed by physicians and patients themselves. Among the physician-assessed ESAS scores, the highest mean score was reported for pain (5.07 ± 2.58), while the lowest was reported for dyspnea. Similarly, in the patient's group, the highest mean score accounted for pain (5.45 ± 2.83), whereas the lowest was reported for nausea [5]. Results of a study by Delgado-Guay et al. also reported a high median score of five for pain and fatigue, and the lowest score of 0 for nausea and dyspnea [15].

Wangnamthip et al. compared the ESAS scores at baseline and the third visit in their study, with the highest median score of five for fatigue at baseline, which was reduced to a median of 4.5 at the third visit. The lowest median score of 0 was reported for nausea and depression at both baseline and the third visit. The total median score was 27.5 at baseline, which decreased to 23.5 at the third visit [16]. Similarly, the results of a study by Hui et al. demonstrated that the total ESAS score of 31.2 at the first visit was reduced to 28.5 at the second visit of patients [7]. Furthermore, a study by Azhar et al. compared the ESAS scores among the scheduled and unscheduled patient groups. The highest median score accounted for fatigue in both groups, while the lowest median score was recorded for nausea in both groups. In the unscheduled group for follow-up visits, the highest median score of seven was recorded again for fatigue. However, among the scheduled group of follow-up patients, a slightly lower median score of five was reported, while the lowest median score was reported for nausea in both groups of follow-ups [14]. These results are presented in Table 3.

**Table 3:** Edmonton Symptom Assessment System (ESAS) scores reported in the included studies NR: Not reported.

Study	Value	Symptoms domain												
		Pain	Fatigue	Nausea	Depression	Anxiety	Drowsiness	Lack of appetite	Well-being	Dyspnea	Insomnia	Total symptom distress score	Emotional/psychological distress score	Physical distress score
Shin et al. [11]	Mean (95% CI)	5.1 (4.8-5.4)	6.5 (6.2-6.7)	2.2 (2.0-2.5)	3.3 (3.0-3.5)	4.0 (3.7-4.3)	4.4 (4.1-4.7)	5.6 (5.3-5.9)	5.3 (5.0-5.5)	5.3 (5.0-5.5)	4.5 (4.3-4.8)	NR	NR	NR
Yokomichi et al. [12]	Mean ± SD	1.7 ± 2.4	2.0 ± 2.6	0.5 ± 1.4	1.5 ± 2.4	1.7 ± 2.5	1.6 ± 2.4	1.9 ± 2.9	3.1 ± 2.7	1.0 ± 2.0	NR	15 (15)	NR	NR
Kim et al. [13]	Median (IQR)	5 (2-8)	6 (4-8)	1 (0-3)	2 (0-5)	3 (0-5)	4 (1-6)	5 (3-7)	5 (3-7)	3 (0-5)	5 (2-7)	34 (23-46)	5 (1-9)	24 (15-32)
Hui et al. [7]	1st visit	NR	NR	NR	NR	NR	NR	NR	NR	NR	NR	31.2 (16.5)	5.7 (5.5)	21.0 (11.2)
	2nd visit	NR	NR	NR	NR	NR	NR	NR	NR	NR	NR	28.5 (17.4)	5.2 (5.3)	19.2 (11.8)
Azhar et al. [14]	Scheduled; Median (IQR)	5 (2-7)	6 (4-8)	1 (0-3)	2 (0-5)	3 (0-5)	3 (0-6)	5 (2-7)	5 (3-7)	2 (0-5)	5 (3-7)	33 (22, 46)	5 (0, 10)	27 (18, 37)
	Unscheduled; Median (IQR)	7 (5-9)	7 (5-8)	2 (0-5)	3 (0-6)	3 (0-6)	5 (1-7)	5 (2-8)	6 (4-8)	3 (0-5)	6 (4-8)	40.5 (29.5, 52)	6.5 (2, 12)	33.5 (25, 44)
	FU patients scheduled; Median (IQR)	4 (2-7)	5 (3-8)	1 (0-3)	1 (0-4)	2 (0-5)	3 (1-6)	5 (2-7)	5 (3-6)	3 (0-6)	4 (2-6)	30 (19, 42)	4 (0, 8)	26 (21, 45)
	FU unscheduled median (IQR)	6 (4-8)	7 (4-9)	2 (0-5)	3 (0-5)	4 (1-6)	5 (1-6)	6 (3-8)	6 (4-8)	3 (0-6)	5 (3-5)	42 (25, 56)	7 (2, 11)	34 (21, 45)
Brooks et al. [5]	Patient; Mean ± SD	5.45 ± 2.83	5.27 ± 2.70	2.34 ± 2.87	3.17 ± 2.87	4.04 ± 2.91	4.22 ± 2.89	3.47 ± 3.03	4.13 ± 2.54	2.36 ± 2.73	3.51 ± 3.10	NR	NR	NR
	Physician; Mean ± SD	5.07 ± 2.58	5.01 ± 2.59	2.17 ± 2.66	3.31 ± 2.81	4.09 ± 2.82	3.35 ± 2.51	3.29 ± 2.92	4.23 ± 2.48	2.09 ± 2.49	3.31 ± 2.91	NR	NR	NR
Delgado-Guay et al. [15]	Median (IQR)	5 (2-8)	5 (2-8)	0 (0-5)	3 (0-7)	3 (0-6)	3 (0-6)	3 (0-6)	4 (1-6)	0 (0-5)	3 (0-6)	NR	NR	NR
Wangnamthip et al. [16]	Baseline; Median (IQR)	4.0 (2.5–6.0)	5.0 (2.0–6.0)	0.0 (0.0–3.0)	0.0 (0.0–3.0)	3.0 (0.0–5.0)	3.0 (0.5–5.0)	4.0 (0.0–7.0)	4.5 (0.0–6.5)	2.0 (0.0–5.0)	NR	27.5 (17.0–43.0)	NR	NR
	3rd visit; Median (IQR)	3.0 (1.0–5.5)	4.5 (2.0–6.0)	0.0 (0.0–1.5)	0.0 (0.0–2.5)	3.0 (0.0–5.0)	2.0 (0.0–5.5)	2.5 (0.0–5.0)	3.0 (0.0–6.0)	1.0 (0.0–5.0)	NR	23.5 (10.5–36.5)	NR	NR

Higher rates of pain, fatigue, nausea, and insomnia were reported among patients admitted from emergency centers compared to transferred inpatients [11]. A direct correlation between poorer performance status and increased symptom burden was observed by Yokomichi et al., which significantly impacted the patient's quality of life [12]. Similarly, a study by Kim et al. also suggested a correlation between performance status and the symptom burden of patients [13]. The study results by Hui et al. demonstrated that the average improvement between the first and second clinic visits was 1.8 points for physical symptoms, 0.5 points for emotional symptoms, and 2.7 points for total symptom burden [7]. Azhar et al. stated that unscheduled new patients and unscheduled follow-up patients had significantly worse symptom burden, particularly related to pain, fatigue, nausea, depression, anxiety, drowsiness, sleep disturbances, and overall well-being. Higher pain scores were associated with an increased risk of unscheduled visits (OR = 1.18, 95% CI = 1.10, 1.27, p < 0.001) [14]. However, Wangnamthip et al. noted that pain interference diminished at all visits relative to baseline (p < 0.05) [16].

Brooks et al. considered ESAS a beneficial tool for establishing symptom control priorities and guiding appointments [5]. Wangnamthip et al. described that more than 80% of the patients were satisfied with cancer pain management in the clinic, and their satisfaction increased to 88.9% on the third visit [16]. Overall, the results suggest that the ESAS scale not only helps in assessing the symptom burden at that point but can also aid in determining the symptom burden over time. Only two studies included in this review reported satisfaction with the ESAS tool, while all other studies did not assess or report satisfaction levels, either from a patient's perspective or from a physician's perspective. These results in detail are represented in Table 4.

**Table 4:** Comparative overview of symptom burden, quality of life, and healthcare provider satisfaction in palliative care patients assessed using ESAS score ESAS: Edmonton Symptom Assessment System; PGI: Patient's Global Impression Scale; FU: Follow-up; M(IQR): Median interquartile range; EC: Emergency center; OR: Odds ratio; ECOG PS: Eastern Cooperative Oncology Group performance status; PC: Palliative care; RT: Radiotherapy; NR: Not Reported; NRS: Numerical Rating Scale.

Study	Overall symptom burden	Patient's quality of life	Satisfaction
				Shin et al. [11]	EC patients have higher rates of pain, fatigue, nausea, and insomnia.	NR	NR
Yokomichi et al. [12]	The total ESAS-r score is significantly higher in patients with performance status 2-4 compared to 0 and 1 (P < 0.0001).	Patients with ECOG performance status of 2 or more had a significantly higher symptom burden in six categories compared to those with a performance status of 0 and 1, indicating a direct correlation between poorer performance status and increased symptom burden, thus impacting overall quality of life.	NR
Kim et al. [13]	PC specialists’ assessments correlated with ESAS fatigue, dyspnea, anorexia, feeling of well-being, and symptom distress scores. Higher symptom burden tended to have higher ECOG PS scores, suggesting a correlation between overall symptom burden and performance status.	Symptom distress score M(IQR): 34 (23-46), physical distress score M(IQR): 24 (15-32), psychological distress score M(IQR): 5 (1-9)	
Hui et al. [7]	The average improvement between the first and second clinic visits was 1.8 points for physical symptoms, 0.5 points for emotional symptoms, and 2.7 points for total symptom burden.	Patient-reported outcomes were collected using the PGI during the second clinic visit. Approximately 45% of patients reported their physical symptoms as "better," 28% reported emotional symptoms as "better," and 37% reported total symptom burden as "better."	NR
Azhar et al. [14]	Unscheduled new patients and unscheduled patients had significantly worse symptom burden, particularly related to pain, fatigue, nausea, depression, anxiety, drowsiness, sleep disturbances, and overall well-being. This suggests that unscheduled patients, both new and follow-up, experienced a higher overall symptom burden compared to scheduled patients. Higher pain scores were associated with an increased risk of unscheduled visits (OR = 1.18, 95% CI = 1.10, 1.27, p < 0.001).	Unscheduled new patients: M(IQR) = 40.5 (29.5, 52), emotional (0.006), physical (<0.001), and total scores (<0.001) were also significantly higher.	NR
Brooks et al. [5]	NR physicians tend to rate psychological and global symptoms slightly more.	NR	A beneficial tool for establishing symptom control priorities and guiding appointments. Presents challenges in completing the ESAS but is overall helpful. Participants reported satisfaction with the ESAS tool.
Delgado-Guay et al. [15]	NR	High importance is placed on spirituality/religiosity in coping with illness, strength, comfort, and positive well-being.	NR
Wangnamthip et al. [16]	150 newly referred cancer patients enrolled, and 72 completed all three follow-ups (48%). Of these, 61% achieved a favorable response at FU3. Pain interference diminished at all visits relative to baseline (p < 0.05).	Palliative care referrals had higher initial NRS and lower performance status compared to those who completed follow-ups.	Patients were satisfied with cancer pain management in the clinic by more than 80% at every visit and reached 88.9% on the third visit.

Discussion

First published in 1991, the ESAS was intended to be a tool for daily symptom audits for patients in palliative care units. This one-page screening tool is widely regarded as useful, dependable, and valid. Its simplicity and brevity have been highly acclaimed [17]. This review explored the importance of ESAS among palliative care patients in terms of symptom assessment and burden. The findings exhibited that fatigue followed by pain were the symptoms with the highest score on ESAS among all the studies, while the lowest score was recorded for nausea and dyspnea in the majority of the studies.

Similarly, results of a study by Verhoef et al. reported that patients prioritized pain (26%), fatigue (9%), and shortness of breath (9%), with fatigue and loss of appetite (median = 7) accounting for the most burden [18]. Results of another study by Mitchell et al. also showed that the most common and severe symptoms were fatigue (86% of visits; ESAS ≥ 4 in 55% of visits), poor appetite (69%; ESAS ≥ 4 in 42%), and a low sense of well-being (78%; ESAS ≥ 4 in 38%) [19]. Shamieh et al. narrated that one recurring issue experienced by female patients with breast cancer was fatigue. It intensified significantly after diagnosis and persisted for over six months, gradually decreasing in intensity over time, and it may continue for years following treatment [20].

Results of a retrospective review reported that pain, fatigue, and drowsiness were the most common symptoms upon presentation (>50%), and the two most severe symptoms, with median ESAS values of seven (on a scale of 0-10), were pain and fatigue [21]. The results of a prospective study showed that among the recorded symptoms, loss of appetite accounted for 92.73%, while nausea was the least common (54.55%). Pain was the most prevalent and distressing symptom, as stated by 40% of patients, while 64.55% of patients reported experiencing one or more symptoms that were severe enough to keep them from sleeping [22]. Similarly, findings of a population-based study demonstrated that almost 50% of patients had moderate-severe symptoms of fatigue, loss of appetite, and lack of well-being earlier in the disease course (weeks 18 to 12 before death), while 50% had moderate-severe symptoms of drowsiness, pain, and shortness of breath later in the disease course (weeks five to two before death). In every dimension, outpatient palliative care was linked to a higher probability of moderate-to-severe scores, with pain receiving the highest score [23]. These findings from the literature are in accordance with our results. However, in our study, fatigue scores were the highest, and only two studies reported the highest score for pain.

In this systematic study, it was observed that over the follow-up appointments or visits, ESAS scores improved for the better. Similarly, Lee et al. stated that patients with moderate/severe symptom burden (ESAS ≥ 5; 6.5 at baseline; 4.5 at the first follow-up; 3.6 at the second follow-up; p < .001) showed a significant improvement in their symptoms [24]. Likewise, Rafaqat et al. reported that the two visits resulted in a statistically significant improvement in pain (5.0 vs. 2.5, p < 0.001), loss of appetite (5.0 vs. 4.0, p = 0.004), depression (2.0 vs. 0.0, p < 0.001), and anxiety (1.5 vs. 0.0, p = 0.032) as well as an overall decrease in ESAS scores (6.0 (2.8, 11.0), p < 0.001). Hence, the authors suggested that for individuals with a terminal diagnosis, an outpatient visit for palliative care dramatically reduced their symptom burden [25].

Also, the results of a study by Shamieh et al. showed that over time, there was a significant improvement in both ESAS pain (5.9 vs. 5.1, P = 0.004) and sleep (4.6 vs. 4.1, P = 0.007). Pain, fatigue, nausea, depression, anxiety, drowsiness, appetite, well-being, dyspnea, and sleep all showed significant improvements among patients who initially presented with moderate-to-severe symptoms. The intensity of the remaining ESAS symptoms diminished; however, this difference was not statistically significant [26]. Similarly, Cervantez et al. reported no statistically significant differences in physical and emotional symptom sub-scores, as well as the overall symptom burden, when patients' symptom scores were assessed during follow-up visits with their oncologists [27]. Diplock et al. described a strong relationship between ESAS change and quality of life, suggesting that ESAS may be a valuable tool for alerting medical professionals to symptoms and functional changes that may occur between visits [28].

Selby et al. narrated that in palliative care literature, terms such as symptom burden and symptom distress are commonly encountered and employed in many contexts, encompassing the augmentation of symptom scores and comprehensive evaluations of function disruption. Although prior evaluations have indicated the significance of such input, patient feedback has not played a significant part in these diverse definitions to date. A group of patients with a high degree of self-defined burden are those with advanced disease, followed by palliative care professionals. Additionally, a patient who scores one or more symptoms on the ESAS ≥ 7 is likely to be significantly impacted in terms of their physical, emotional, and social functioning, and they are at a high risk of developing a self-defined burden. Subsequent research ought to examine which symptom management strategies best facilitate a reduction in perceived burden [29]. Moreover, McGee et al. suggested an innovative use for the ESAS as a prognostic tool that could supplement current performance status and patient evaluation models in the creation of the best possible treatment regimens and survival estimates for patients with advanced lung cancer [30]. Two of the studies in our review observed a correlation between performance status and symptom burden.

Schulman-Green et al. described certain advantages and challenges associated with the utilization of ESAS in palliative care settings. The ESAS had the advantage of being an easy-to-use instrument that could identify concerned issues, include patients in symptom evaluation, and track changes in symptoms over time. Furthermore, the ESAS was thought to be a helpful teaching tool for staff members with less expertise. Lack of clarity on inclusion criteria and assessment frequency was one of the challenges. Other issues included difficulties understanding the numerical symptom rating scale, integrating patient preferences with symptoms, and the perception that using standard assessment tools was unnatural [31]. Additionally, Wong et al. stated that the belief that symptom assessment could be time-consuming is a primary cause for its non-implementation. However, the median time to complete an ESAS form for patients with advanced cancer who had never done so was less than two minutes, consisting of 73 seconds for self-filling and 109 seconds for aided completion [8].

Further stressing the advantages of using ESAS, the study by Sutradhar et al. reported that a 51% reduction in the risk of death was shown to be substantially correlated with overall ESAS exposure (HR = 0.49, 95% CI = 0.48-0.50, p-value < 0.0001) [32]. Findings of a study by Goyal et al. concluded that using ESAS during on-treatment visits was linked to stable or improved symptom severity in cases where more easily accessible therapeutic interventions, like counseling, painkillers, anti-emetics, appetite stimulants, and anti-anxiolytics, are available. Using validated patient-reported symptom-scoring instruments could help with provider management [33]. Two of the included studies in this review showed significant satisfaction with the utilization of ESAS.

Our review is one of the few systematic reviews assessing the symptom burden using ESAS in palliative care settings, to the best of our knowledge. The systematic search methodology and the analysis of all keywords in this field are the main strengths of this study. However, the inclusion of studies with serious and critical risk of bias and observational studies are the limitations of this study. Additionally, considering only English papers and the specified publication period are further limitations of this study.

## Conclusions

ESAS plays a vital role in assessing and managing symptom burden in palliative care patients, ultimately improving their overall quality of life. It assists healthcare professionals and family caregivers in methodically assessing and treating symptoms over time. By fostering better communication among care teams and involving caregivers in the process, the use of ESAS promotes a more patient-centered approach to care, ensuring that both the patient's needs and the perspectives of their loved ones are considered. Further research, including randomized trials with more robust study designs, is needed to assess and compare the influence of ESAS on the symptom burden of various spectrums of diseases as well as to assess physician and patient satisfaction levels with its use.
